# Dispersion coding of ENZ media via multiple photonic dopants

**DOI:** 10.1038/s41377-022-00892-8

**Published:** 2022-07-06

**Authors:** Ziheng Zhou, Hao Li, Wangyu Sun, Yijing He, Iñigo Liberal, Nader Engheta, Zhenghe Feng, Yue Li

**Affiliations:** 1 Department of Electronic Engineering, Tsinghua University, 100084 Beijing, China; 2 Department of Electrical and Electronic Engineering, Public University of Navarre, Pamplona, 31006 Spain; 3 Department of Electrical and Systems Engineering, University of Pennsylvania, Philadelphia, PA 19104 USA

**Keywords:** Metamaterials, Nanophotonics and plasmonics

## Abstract

Epsilon-near-zero (ENZ) media are opening up exciting opportunities to observe exotic wave phenomena. In this work, we demonstrate that the ENZ medium comprising multiple dielectric photonic dopants would yield a comb-like dispersion of the effective permeability, with each magnetic resonance dominated by one specific dopant. Furthermore, at multiple frequencies of interest, the resonant supercouplings appearing or not can be controlled discretely via whether corresponding dopants are assigned or not. Importantly, the multiple dopants in the ENZ host at their magnetic resonances are demonstrated to be independent. Based on this platform, the concept of dispersion coding is proposed, where photonic dopants serve as “bits” to program the spectral response of the whole composite medium. As a proof of concept, a compact multi-doped ENZ cavity is fabricated and experimentally characterized, whose transmission spectrum is manifested as a multi-bit reconfigurable frequency comb. The dispersion coding is demonstrated to fuel a batch of innovative applications including dynamically tunable comb-like dispersion profiled filters, radio-frequency identification tags, etc.

## Abstract (web summary)

The multiple photonic dopants contained in an epsilon-near-zero medium can serve as non-interacting resonators, and offer an opportunity for the independent control of electromagnetic waves at various frequencies.

## Graphical abstract

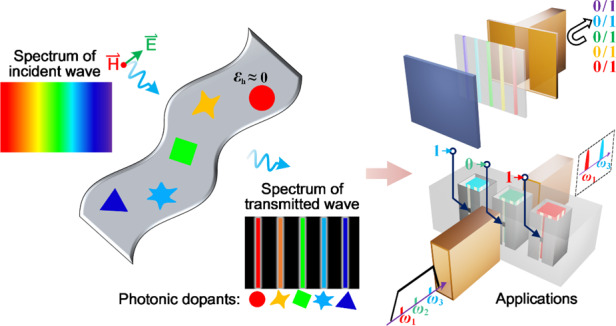

## Introduction

Media with extremely small permittivity, i.e., the epsilon-near-zero (ENZ) media^1–6^, have drawn great interest from the fields of physics and material science. Due to their peculiar constitutive parameter, wave in the ENZ media features an extremely stretched wavelength and an infinite phase velocity, which enables a temporal dynamics of field with static spatial distribution. ENZ media have been exploited to demonstrate many exotic wave phenomena as well as advanced functions, such as supercoupling of wave energy through arbitrary shaped narrow channel^1,2^, flexibly shape scattering/radiation patterns^7–12^, boosting optical nonlinearity^13,14^, and trapping light in three-dimensional nanostructures^15,16^, perfect coherent absorption^17–19^, just to name only a few. In the latest years, the emerging technique of photonic doping^20^ opens a pathway to flexibly tailoring the behaviors of the ENZ medium interacted with wave. In this scheme, via an arbitrary located dielectric impurity, ones are able to alter the magnetic field configuration over the whole ENZ host, attaining either epsilon-and-mu-near-zero (EMNZ) medium^21–24^ or the perfect magnetic conductor (PMC) body with an infinite permeability.

In this work, by leveraging photonic dopants as metamaterial bits, we propose the concept of the dispersion coding, with the aim to sculpt the spectral response of the composite material in a discrete and reconfigurable fashion. We demonstrate that a single photonic dopant can be uniquely characterized by its own resonance signature, which manifests as a zero closely followed by a pole in the effective permeability function. Once multiple different impurities are doped in the ENZ host, a series of resonant signatures with slight frequency offsets can be synthesized in the spectrum of effective permeability, giving rise to a comb-like dispersion^25–27^. As a nontrivial property, each pole in the effective permeability of doped ENZ medium is explicitly determined by the dopant resonating at this frequency; and multiple photonic dopants in this circumstance behave as noninteracting resonators while still being coupled to external excitations, which are rarely the case for the densely packed resonator systems^28,29^. Therefore, the analog spectrum response of the material can be independently quantified as multi-bit states at a series of frequencies, dependent on whether corresponding dopants are included nor not. Inspired by this, we introduce the methodology of discrete coding to the doped ENZ medium―harness the presence or absence of each dopant as binary states to program the permeability dispersion of the material, and the behavior of multi-doped ENZ material under illumination, being opaque or transparent, allows to be controlled discretely in the spectrum.

The proposed concept of dispersion coding is distinct from the previous metamaterial coding schemes. For example, in the spatial coding^30–34^ of metamaterials, the specially arranged meta-atoms were explored for wavefront manipulations, which can be controlled by the field programmable gate arrays^31^ and artificial intelligence algorithms^34^. In this work, rather than focusing on the spatial modulation of wave, we extrapolate the philosophy of discrete coding to a different dimension—frequency domain, to program the response of the medium at multiple frequencies. As an essential difference from the frequency coding scheme of metamaterials as proposed in the previous work^35^, the dispersion coding of doped ENZ medium is anomalously independent of the spatial arrangement and order of the photonic dopants. The ENZ host seems to effectively eliminate the spatial correlation of the photonic dopants, and the dopants can be located following arbitrary arrangements or even randomly. Such a spatial-arrangement irrelevant essence is in contrast to the conventional metamaterials with periodic or quasi-periodic structures^36–38^, and thus the shape as well as the layout of the doped ENZ medium can be arbitrarily chosen. Furthermore, the absence of mutual coupling among multiple photonic dopants can offer an exciting opportunity for independent dispersion engineering at various frequencies.

For experimental verification, a waveguide-emulated plasmonic medium^39^ is exploited to emulate the ENZ host, and multiple dopants are fabricated from ceramic blocks. The measured comb-shape profile of transmission spectrum is in agreement with our theory. We also verify the efficient approach to the control of both number and position of the markers in the frequency comb via choosing dielectric photonic dopants. The dispersion coding can inspire a series of powerful applications. A reconfigurable comb-like dispersion profiled filter is proposed for smart signal processing. A novel frequency division multiplexing system and a tag suitable for radio-frequency identification are also envisioned and numerically verified, based on the platform of multi-doped ENZ material. These designs can be extended from microwave region to optical frequencies, where suitable ENZ materials can be found^3,40^. Therefore, the proposed dispersion coding of doped ENZ medium provides a new perspective on the far-reaching concept of digital artificial material, opening exciting horizons for applications in material science, communication engineering, terahertz/optical regime, etc.

## Results

### Concept and theoretical analysis

**Fig. 1:**
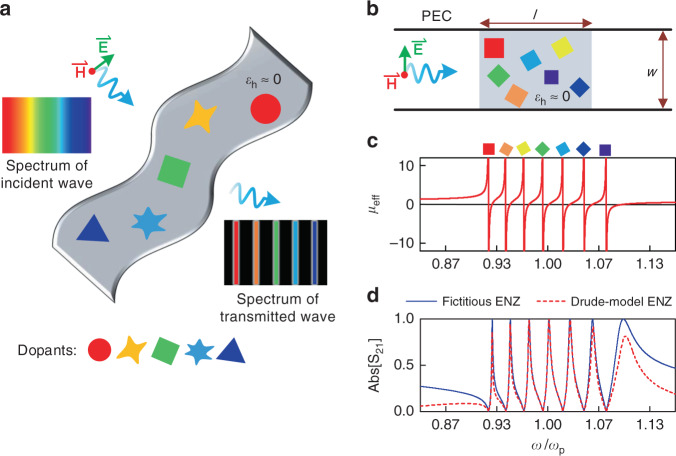
Multiple dopants in ENZ medium as noninteracting resonators. **a** Conceptual sketch of a two-dimensional (2D) ENZ host with a relative permittivity ε_h_ ≈ 0 containing multiple arbitrarily-located dielectric rods as “dopants”, which act as noninteracting resonators filtering the light at their individual resonant frequencies. **b** Schematic plot of the two-dimensional (2D) ENZ medium in which seven dopants with square cross sections are embedded. Geometry parameters l and w are λp and 0.8λp, respectively, where λp is the free-space wavelength at ωp =2π × 3 × 10^9^ rad/s. The dopants are chosen with a relative permittivity ε_d_ of 37 and slightly different side lengths. The incident wave is TM-polarized with a magnetic field along the out-of-the-plane axis. **c** Calculated relative effective permeability and (**d**) transmission amplitude for the seven-dopant configuration in (**b**) under conditions of fictitious nondispersive ENZ and Drude-dispersive ENZ hosts

The idea of leveraging multiple photonic dopants to engineer the transmission response of an ENZ medium at multiple frequencies is conceptually illustrated in Fig. 1a, where several dielectric impurities acting as photonic dopants are arbitrarily arranged in a 2D host whose relative permittivity *ε*_h_ is close to zero. For the incidence of transverse-magnetic (TM) polarized wave, the embedded photonic dopants serve to impact the magnetic field distribution over the whole 2D ENZ host, and the effective permeability of the whole medium allows for a fine control over the range from zero to infinity^20^. For the frequencies where the effective permeability of the doped ENZ slab is close to zero, i.e., EMNZ medium, the supercoupling phenomenon with high transmission amplitude is observed. On the other hand, at the poles of permeability, i.e., at the resonance of the dielectric dopants, the doped ENZ slab behaves as the PMC body, which is totally opaque for TM-polarized incidence waves. As a result, with the proper design of alternatively distributed permeability zeros and poles, an interesting comb-like transmission spectrum is expected, as schematically depicted in Fig. 1a.

A more specific example used for the theoretical analysis is illustrated in Fig. 1b. A two-dimensional ENZ host is assumed with the relative permittivity *ε*_h_ described by the Drude model, that is *ε*_h_ = 1 − *ω*_p_^2^/*ω*^2^, where *ω*_p_ (= 2π × 3 × 10^9 ^rad/s) is the plasma frequency. Seven square dielectric dopants are chosen with slightly different side lengths, which are 0.126*λ*_p_, 0.123*λ*_p_, 0.120*λ*_p_, 0.117*λ*_p_, 0.114*λ*_p_, 0.111*λ*_p_, and 0.108*λ*_p_ (*λ*_p_ is the wavelength in free space at *ω*_p_). The relative permittivity *ε*_*d*_ (*d* = 1–7) of the dopants is chosen as 37. The doped system is placed in a plate waveguide for evaluation of its transmission performance. The effective relative permeability *μ*_eff_ of the doped ENZ medium can be derived via averaging the magnetic flux over regions of the dopants as well as the ENZ host, which is generally formulated as^20^

1 $$\mu _{{{{\mathrm{eff}}}}} = (A - \mathop {\sum}\nolimits_d {A_d} + \mathop {\sum}\nolimits_d {\mathop {\iint}\nolimits_{A_d} {\psi ^d({{{\mathbf{r}}}}){{{\mathrm{d}}}}s)/A} }$$

where *A* is the cross-sectional area of the whole doped medium, *A*_*d*_ (*d* = 1, 2, 3…) are the cross-sectional areas of the dopants included, while *ψ*^*d*^ (*d* = 1, 2, 3…) denotes the magnetic field in the dopants normalized to unity on their boundaries. Crucially, the contributions from each of the dopants take place in an additive manner, i.e., as if the dopants were not interacting among themselves. This is a unique characteristic of ENZ media, which has not been predicted for other materials that are subjected to conventional effective medium theories.

For rectangular dopants^41^ with areas of *l*_*d*_ × *w*_*d*_ (*d* = 1, 2, 3…), the effective relative permeability of the multi-doped ENZ medium can be explicitly derived as follows:

2 $$\mu _{{{{\mathrm{eff}}}}}(\omega ) \approx {{{\mathrm{1 + }}}}\mathop {\sum}\limits_{d = 1} {\frac{{{{{\mathrm{64}}}}l_dw_d}}{{\pi ^4lw}}\frac{{\omega ^2}}{{\omega _d^2 - \omega ^2}}}$$

Here, *l*×*w* is the cross-sectional area of the ENZ host shown in Fig. 1b, and the poles of the permeability function are expressed by

3 $$\omega _d = \frac{c}{{\sqrt {\varepsilon _d} }}\sqrt {\left( {\pi {{{\mathrm{/}}}}l_d} \right)^2 + \left( {\pi {{{\mathrm{/}}}}w_d} \right)^2}$$

which actually are the eigenfrequencies of the transverse-magnetic TM_11_ (with respect to the out-of-plane axis) mode of the dopants. Detailed derivation of Eq. (2) is presented in Supplementary Note 1. Equation (2) clearly demonstrates that a series of magnetic resonances characterized by diverging effective permeability of the doped medium are gathered in the spectrum. The calculated permeability for the case in Fig. 1b is reported in Fig. 1c. As seen, the permeability function demonstrates an unequivocal comb-shaped dispersion. Importantly, the poles of this distribution are entirely determined by each dopant’s characteristics, and not by the coupling among them. In other words, the frequency associated with the pole induced by one dopant is independent of the dopant position within the host, the presence of other dopants, and the overall shape of the 2D ENZ host. We highlight this relationship by associating each dopant with its corresponding magnetic resonance in Fig.1c. It is also observed from Fig. 1c that the curve crosses zero slightly above each magnetic resonance frequency, where the multi-doped ENZ medium becomes totally transparent for normal TM incidence due to the EMNZ effect.

To quantitatively analyze how multi-doped ENZ medium modulates the wave propagation, we compute the transmission amplitude through the doped ENZ slab shown in Fig. 1b by using the transmission matrix method. In addition, we evaluate the impact of the material dispersion by comparing two cases: A fictitious case of a nondispersive ENZ host having a constant relative permittivity of 0.001 over the frequency band of interest, and a realistic dispersive ENZ host described by Drude model with the plasma frequency *ω*_p_ = 2π × 3 × 10^9 ^rad/s. Detailed information on this calculation is presented in Supplementary Note 2. Figure 1d quantitatively demonstrates a comb-shaped spectral response with transmission peaks and zeros alternatively located at the EMNZ and PMC frequencies. Under the realistic circumstance of a dispersive ENZ host, the transmission amplitude of doped system is reduced for the frequency far away from *ω*_p_, and the frequency comb is observed with an envelope in the amplitude. The dB values of the transmission amplitudes in Fig. 1d are shown in Figure S2.

**Fig. 2:**
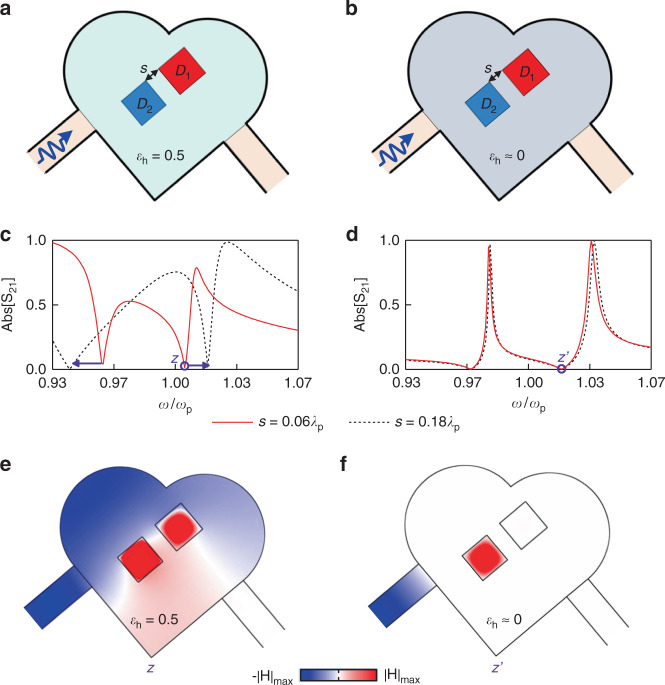
Properties of multiple dielectric rods in the ENZ and non-ENZ hosts. Configurations of (**a)** the two-dimensional (2D) non-ENZ host (*ε*_h_ = 0.5) and (**b**) ENZ host (*ε*_h_ ≈ 0) containing two square dielectric rods. The permittivity of ENZ medium is Drude dispersive, with the angular plasma frequency of *ω*_p_ = 2π × 3 × 10^9 ^rad/s. Host areas in two cases are 0.33*λ*_p_^2^ (*λ*_p_ is the free-space wavelength at *ω*_p_), while square rods *D*_1_ and *D*_2_ are chosen with the permittivity *ε*_*d*_ = 37 and side lengths of 0.122*λ*_p_ and 0.116*λ*_p_, respectively. (**c**) and (**d**) are respectively the simulated transmission spectral response in non-ENZ and ENZ hosts, for different spacing *s* of dielectric rods. (**e**) and (**f**) are snapshots of the magnetic field distribution at the transmission zeros *z* and *z*’ for the cases of the non-ENZ host and ENZ host

To clearly demonstrate that the magnetic resonances of multiple dielectric dopants (rods) in the ENZ medium are independent, we compare the cases of two closely-spaced dielectric rods in the non-ENZ medium (*ε*_h_ = 0.5) and ENZ medium (*ε*_h_ ≈ 0), which are respectively shown in Fig. 2a, b. The permittivity dispersion of ENZ medium is given by the Drude model, with the angular plasma frequency of *ω*_p_ = 2π × 3 × 10^9 ^rad/s. The areas of the hosts in two cases are both 0.33*λ*_p_^2^ (*λ*_p_ is the free-space wavelength at *ω*_p_), while square rods *D*_1_ and *D*_2_ are chosen with the permittivity *ε*_*d*_ = 37 and cross-sectional side lengths of 0.122*λ*_p_ and 0.116*λ*_p_, respectively. The simulated transmission responses for the cases of non-ENZ host and ENZ host are respectively shown in Fig. 2c, d, where the different spacings *s* of dielectric rods are considered. The dB values of the transmission amplitudes in Fig. 2c, d are shown in Fig. S3a, b, respectively. The magnetic resonances of dielectric rods can manifest as zero transmission in the spectral responses. As clearly observed in Fig. 2c, d, the transmission zeros in the non-ENZ-host case move sensitively with the change of *s*, while the transmission zeros in the ENZ-host case are virtually unmoved. The underlying physics is interpreted as follows. Generally, the modes of closely-spaced resonators are spatially coupled, and the variation of resonator spacing can lead to the change of coupling strength, and thereby modifies the resonance frequencies^42,43^. That is actually the case in Fig. 2c. For the doped ENZ medium, however, each magnetic resonance, i.e., the pole of effective permeability (Eq. (3)), are independent and determined solely by one specific dopant. Consequently, the multiple dielectric dopants in the ENZ host can be decoupled at their resonances, which is substantially distinct from the case in the non-ENZ host. To better understand this point, we inspect the magnetic field distributions at the transmission zeros *z* and *z*’, for the cases of the non-ENZ host and ENZ host. In the non-ENZ host case (Fig. 2e), the fields are excited in both rods at the resonance, implies the modes of two rods interact between each other. In contrast, for the case of ENZ host (Fig. 2f), we can stimulate the mode of single dielectric rod at its resonance frequency, which ensures the absence of the interaction among the rods.

**Fig. 3:**
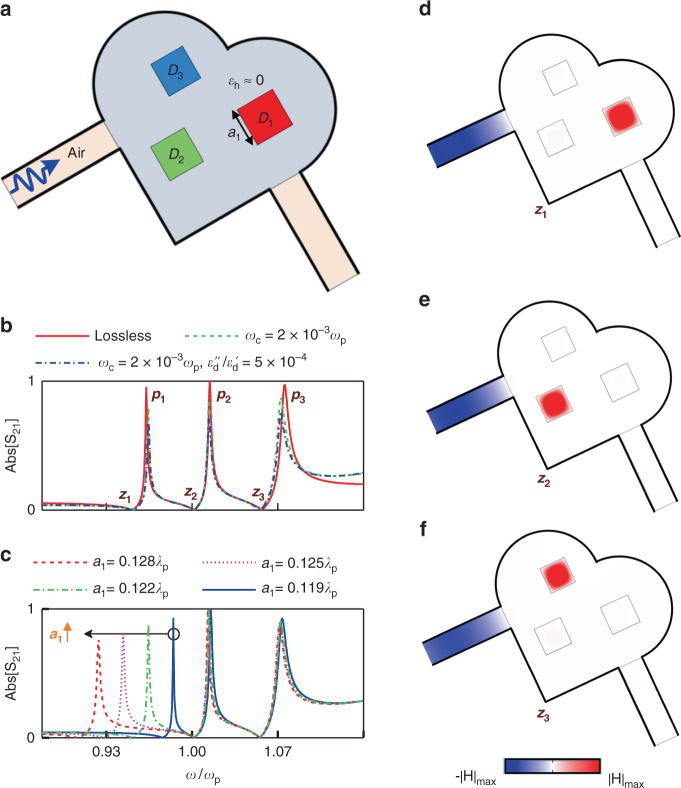
Tunable response and field configuration analysis. **a** Configuration of a 2D ENZ host containing three dopants with square cross sections, which is connected by two air-filled waveguides. The cross-sectional area of the ENZ host is 0.33*λ*_p_^2^ (*λ*_p_ is the free-space wavelength at *ω*_p_ = 2π × 3 × 10^9 ^rad/s), while three square dopants *D*_1_, *D*_2_, and *D*_3_ are chosen with the permittivity *ε*_*d*_ = 37 and side lengths of 0.122*λ*_p_, 0.116*λ*_p_, and 0.11*λ*_p_, respectively. **b** Simulation results for the transmission amplitude spectrum for lossless and lossy cases. **c** Simulation results for the transmission amplitude spectrum for side length of the dopant *D*_1_ changing from 0.119*λ*_p_ to 0.128*λ*_p_ with different values. The snapshots of the magnetic field distribution for the zeros of transmission *z*_1_, *z*_2_, and *z*_3_ are respectively shown in (**d**), (**e**), and (**f**)

Next, we consider the ENZ medium comprising three photonic dopants. That configuration is illustrated in Fig. 3a. The ENZ host is assumed to follow the lossy Drude model with permittivity *ε*_h_(*ω*) =1 − *ω*_p_^2^/(*ω*^2^ + *iω*_c_*ω*), where *ω*_c_ is the collision angular frequency. The square dopants denoted by *D*_1_, *D*_2_, and *D*_3_ have a dielectric constant *ε*_*d*_ of 37 and slightly different side lengths. This two-dimensional configuration is surrounded by perfect electric conductor (PEC). Here we show in Fig. 3b the simulated transmission amplitude under the consideration of losses from the dopant (with a dielectric loss tangent of 5 × 10^−4^) as well as the plasmonic host. As seen, the EMNZ tunneling peaks have a moderate robustness to the materials’ loss. Next, we investigate the interactions among the dopants in the ENZ host, by examining what possible effect tuning of one dopant’s magnetic resonance frequency may have on others’ resonances. As depicted in Fig. 3c, when increasing the dimension *D*_1_, its spectral signature, i.e., transmission peak *p*_1_ and zero *z*_1_, shifts towards lower frequencies, while the transmission zeros contributed by the other photonic dopants are nearly unchanged. Therefore, it can be concluded that dielectric dopants at the PMC resonances are almost uncorrelated, even though their spatial separations could be significantly less than the wavelength. The dB values of the transmission amplitudes in Fig. 3b, c are shown in Figs. S4a, b, respectively.

Analyzing the field distribution over the doped ENZ medium provides additional insight on the physics underlying the effect of those multiple uncorrelated resonances. The simulated snapshots of magnetic field over the lossless doped medium at transmission zeros *z*_1_, *z*_2_, and *z*_3_ are reported in Fig. 3d–f, respectively. At this point, the ENZ host effectively behaves as a PMC medium. Therefore, the magnetic field is confined within one dopant at its magnetic resonance frequency, and vanishes over ENZ host and inside other dopants. In this manner, the magnetic fields in the photonic dopants at these magnetic resonances shall not overlap, which can be a special phenomenon in the ENZ media and underlies the physics behind the absence of interaction among resonators. Under the circumstances associated with transmission peaks, the total magnetic flux in dopants is exactly counteracted the flux in the 2D ENZ host, effectively leading to an EMNZ behavior. In summary, the position of each transmission zero is uniquely defined by one corresponding dopant and hence it is independent of the presence of other resonators. On the other hand, the residual shift of the transmission peaks is due to the additive contributions of the other dopants is verified to be quite tiny, although resonators are densely packed and couple to a same environment. Consequently, the spectral signature of each dopant, characterized by zero transmission followed by a peak, can be designed individually and discretely. This effect enables the independent manipulation of the composite medium’s macroscopic responses at different frequencies. The irrelevance between the transmission response of the doped ENZ medium and spatial arrangement of dopants are presented in Fig. S5. Figures S6, S7 clearly evidence that for the cases of identical and differently-sized dopants, magnetic field distributions in the dopants are independent of their spacing.

Here we can formally define a binary state for the dopant, labeling the case as “1” if a designated dopant is embedded to generate its magnetic resonance signature, or otherwise as “0” to present the absence of that dopant, which manifests as a non-resonant permeability. Assisted by multiple dopants serving as independent bits, one can easily control, in a digital fashion, both the frequencies and the number of multiple magnetic resonance lines in the permeability function (Eq. (2)). This effect can be referred to as dispersion coding, where it is possible to discretely program a material’s constitutive parameters, as well as its macroscopic behavior at various frequencies. It is also worth noting that the multi-doped ENZ medium is inherently different from conventional periodic metamaterials in that its elements, i.e., photonic dopants, can be arbitrarily arranged, while the macroscopic behavior of the whole medium remains unchanged. The independence of the spatial arrangement of the dopants is due to the spatially static-like property of the ENZ host^1,2,20^, in which every location in the host is electromagnetically identical. Aside from its theoretical interest, this characteristic enables the dense integration of noninteracting resonators that at the same time strongly couple to external excitations.

### Experimental verification

**Fig. 4:**
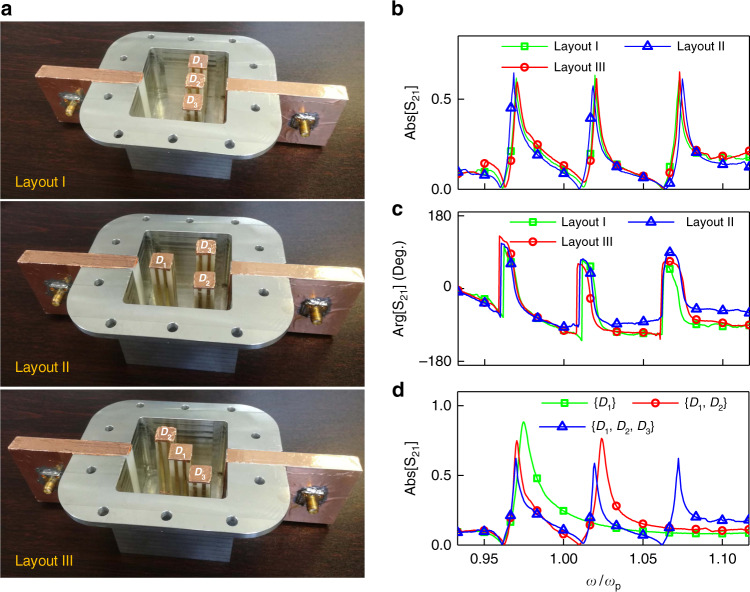
Experimental verification. **a** The photograph of the experimental setup. Three cuboid ceramic blocks (*D*_1_, *D*_2_, and *D*_3_) with permittivity *ε*_1_ = 37 and cross-sectional areas of 0.122*λ*_p_ × 0.122*λ*_p_, 0.116*λ*_p_ × 0.116*λ*_p_, and 0.11*λ*_p_ × 0.11*λ*_p_ (*λ*_p_ is the free-space wavelength at *ω*_p_ = 2π×3×10^9 ^rad/s) are arranged in the metallic cavity with a height *h* of 0.5*λ*_p_ and a cross-sectional area of 0.66*λ*_p_ ×0.5*λ*_p_. The upper plate of the metallic cavity is not shown in the photographs. The doped ENZ cavity is connected to the feeding waveguides filled with Teflon (*ε*_r_ = 2.1). Measured transmission amplitudes (**b**) and phases (after calibration) (**c**) for different spatial arrangements of the dopants in the cavity. **d** Measured transmission amplitude for different combinations of the dopants being used

The experimental verification of the performance of multi-doped ENZ medium as a potential implementation of dispersion coding is carried out with the help of waveguide-emulated plasma^39^. This configuration offers an ENZ condition around the cutoff frequency (*ω*_p_ = *cπ*/*h*) of the waveguide’s TE_10_ mode, where *c* is the light speed in vacuum, and *h* denotes the height of the waveguide. The cross-sectional area of the ENZ host emulated with a metallic waveguide is 0.66*λ*_p_ × 0.5*λ*_p_ (*λ*_p_ is the wavelength in free space at *ω*_p_). The photonic dopants are fabricated with Zirconia ceramic blocks with a relative permeability *ε*_d_ of 37 and a loss tangent of 5 × 10^−4^. The photographs of the assembled prototype with different dopant layouts are shown in Fig. 4a, where two waveguides filled by the Teflon (*ε*_r_ = 2.1) are connected to the doped ENZ cavity for testing the transmission rate. To diminish the undesired excitation of TM modes^20^, the dopants are fenced with thin copper strips placed apart by 0.03*λ*_p_, and in this sense, the entire structure of a dielectric rod fenced by metallic strips is considered as a photonic dopant. Detailed information about the experimental setup can be found in “Materials and Methods”.

The measured transmission amplitudes for three dopants arranged following three different layouts are reported in Fig. 4b, and the results in dB are shown in Fig. S8a. A comb-like spectral transmission response, with alternatively located high-quality-factor tunneling peaks and zeros, is evidenced. Additionally, the transmission spectra of multi-doped systems with different layouts I-III yield are almost the same, which verifies the aforementioned independence of spatial order and locations of dopants. Note that the different layouts include densely packaged resonators, in which the separation between the resonators is much smaller than the wavelength in free space at *ω*_p_. We also measured transmission phases under different cases, and the results are reported in Fig. 4c, where the phase variations in the feeding waveguides have been de-embedded in the calibration procedure. As can be seen, zero phase delays are consistently observed at the tunneling frequencies, which corroborates a series of EMNZ supercoupling are gathered in the frequency domain. As anticipated, the comb-shaped response can be discretely reconfigured via removing or otherwise retaining specific dopants. As a demonstration of this feature, we exclude the dopant *D*_3_ with the smallest size to remove the tick of the highest frequency in the comb, or retain the lowest resonant transmission peak via using a single dopant *D*_1_ with the largest size. The results are clearly demonstrated in Fig. 4d, and their dB values are shown in Figure S8b. As the zero of the effective permeability (Eq. (2)) is not solely determined by one specific dopant, a minor frequency shift of the lowest EMNZ supercoupling peak was observed when dopant combinations were changed. By this simple operation of photonic dopants, one is able to program the dispersion of the whole medium’s response to the external field. Owing to the compact cavity structure and the state-of-the-art low-loss ceramic material, this experimental platform compared with those in the previous work^20^ is much more advantageous to system integration and realistic applications.

### Multi-frequency dynamic filtering

**Fig. 5:**
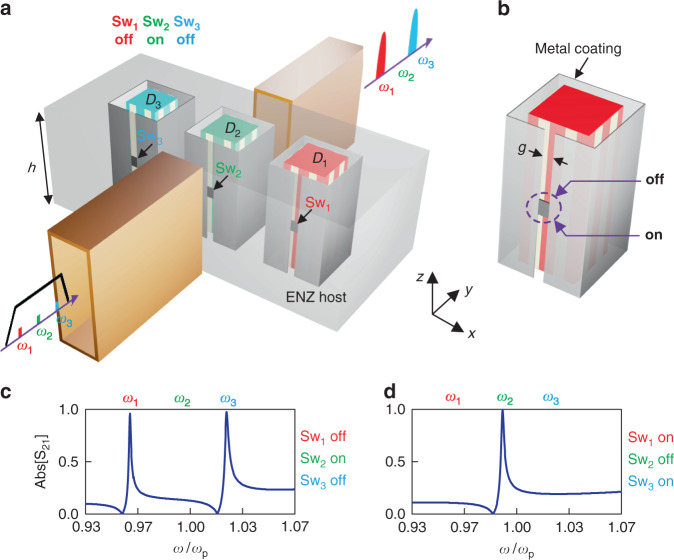
Multi-frequency dynamic filtering. **a** The architecture and working principle of the proposed three channel dynamic filter, where multiple dopants controlled by switches independently govern narrow channels at angular frequencies *ω*_1_, *ω*_2_, and *ω*_3_. The metallic cavity is designed with a height *h* of 0.5*λ*_p_ (*λ*_p_ is the free-space wavelength at *ω*_p_ = 2π × 3 × 10^9^ rad/s). Side lengths of square cross-sections of the dopants *D*_1_, *D*_2_, and *D*_3_ are 0.112*λ*_p_, 0.106*λ*_p_, and 0.097*λ*_p_, respectively. The metallic side walls of the doped cavity are not shown here. (**b**) Configuration of a photonic dopant assembled with a switch. (**c**) and (**d**) are the simulation results for the transmitting spectra under different states of the switches

Dispersion coding enabled by noninteracting photonic dopants pave the pathway to various applications of dispersion engineering and data storage. In this example, we would like to discuss the application of the dispersion coding for a filter with binary-tunable comb-like dispersion. Figure 5a illustrates the proposed architecture of multi-frequency dynamic filtering implemented by noninteracting resonators. Three cuboid dopants *D*_1_, *D*_2_, and *D*_3_ in the waveguide-emulated ENZ environment are chosen with the relative permittivity of 37 and slightly different cross-sectional sizes. The height *h* of the cavity along the *z*-axis is set as 0.5*λ*_p_, which determines the cutoff frequency for the ENZ condition. The cross-sectional area (on the *x–y* plane) of such an ENZ cavity is *λ*_p_ × 0.5*λ*_p_. To enable a dynamical switch of the doped system, each dopant is cloaked by a ring-shaped metallic wall, with a gap of *g* = 0.01*λ*_p_ etched on one face, and then three switches (Sw_1_, Sw_2_, and Sw_3_) are armed across these gaps. The switches can be considered to be either electronic switches in microwave frequencies, or phase change materials, such as vanadium oxides^44^ in the optical frequencies. Details of the structure of a dopant assembled with a switch are shown in Fig. 5b. In practice, the control lines of the switch should adhere to the metallic coating of the dopant, to avoid the unexpected impact on the resonant mode of the cavity.

For the switch being turned off, the photonic dopant in the coated region is normally excited, producing an extremely narrow bandpass response, i.e., the EMNZ supercoupling, at the corresponding frequency. On the other hand, when the switch is turned on, it shorts the gap and “deactivates” the dopant within the closed metallic wall, reducing the tunneling rate, namely the amplitude of the transmission, to almost zero. Numerical studies on magnetic field configurations for a switch being turned off and on are gathered in Figure S9. As shown in Fig. 5a, three narrow frequency channels at angular frequencies *ω*_1_, *ω*_2_, and *ω*_3_ are governed by noninteracting and independently switchable dopants *D*_1_, *D*_2_, and *D*_3_. The simulated transmission spectrum of the proposed dynamic filter for Sw_1_, Sw_2_, and Sw_3_ respectively being at “off”, “on”, and “off” states are shown in Fig. 5c; while the results for Sw_1_, Sw_2_, and Sw_3_ respectively being “on”, “off”, and “on” are shown in Fig. 5d. As expected, the highly-selective transmission responses are precisely generated at the frequencies where the switches of corresponding dopants are “off”. Overall, by this architecture of multiple noninteracting dopants, we accomplish a dynamic frequency-comb filter with high quality factors. The dB values of the transmission amplitudes in Fig. 5c, d are shown in Fig. S10a, b, respectively.

### Radio-frequency tagging

**Fig. 6:**
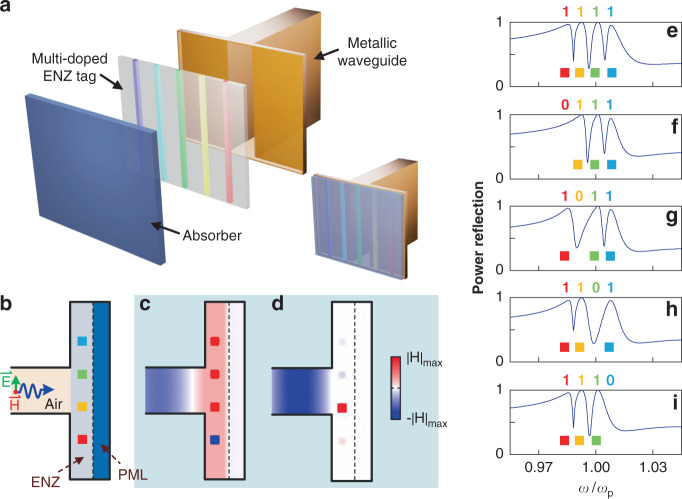
Radio-frequency tagging based on dispersion coding in ENZ structure. **a** Schematic view of the proposed radio-frequency tag inspired by the ENZ-based dispersion coding. Inset: assembled tagging device. **b** The two-dimensional configuration of the proposed tagging device. The ENZ medium has an area of 0.25*λ*_p_ × 2.27*λ*_p_ (*λ*_p_ is the free-space wavelength at *ω*_p_, chosen as 2π × 5.05 × 10^9^ rad/s in this example), while four dopants shown have slightly different cross-sectional sizes of 0.117*λ*_p_ × 0.117*λ*_p_, 0.117*λ*_p_ × 0.114*λ*_p_, 0.117*λ*_p_ × 0.112*λ*_p_, and 0.117*λ*_p_ × 0.110*λ*_p_. A perfect match layer (PML) is used to emulate the absorber in numerical simulation. The snapshot of the simulation results of the magnetic field distribution at (**c**) 0.985*ω*_p_ (a reflection dip) and (**d**) 0.991*ω*_p_ (a reflection peak). **e** The simulation results of reflection spectrum of the configuration shown in (**b**) with four dopants. The reflection spectra for the configuration shown in (**b**) yet with the red, yellow, green, or blue dopant being removed from the ENZ host are shown in (**f)**, (**g)**, (**h**), and (**i**), respectively

Another interesting functionality of the dispersion coding is data storage. We explore this application by designing a radio-frequency tag, which can find promising applications in the fields of multi-frequency sensing, target tracking, and radio-frequency identification (RFID). In our technique, information is simply stored in the tag^15,45,46^ comprising multiple photonic dopants, and the inherent resonance spectrum of the doped ENZ medium structure is regarded as its identification. The perspective view of the proposed design is illustrated in Fig. 6a. A thin layer of doped ENZ layer serves as the tag, which is sandwiched between an absorber and the feeding waveguide when testing. To explain the operating principle, we numerically simulate the 2D structure of the proposed tagging device in Fig. 6b, where ENZ host is described by the Drude model with the plasma frequency *ω*_p_ and four rectangular dopants are set with the slightly different sizes. The relative permittivity of the dopants is still chosen as 37. As expected, the multi-doped ENZ medium enables a series of reflections peaks and dips in the spectrum, and we show the simulated magnetic field for the reflection dip at 0.985*ω*_p_ and the peak at 0.991*ω*_p_ in Fig. 6c, d, respectively. For the EMNZ state shown in Fig. 6c, most power is transmitted through the tag and then dissipated in the absorber. On the other hand, the perfect magnetic conductor state of the tag leads to a strong reflection of power, as shown in Fig. 6d. The reflected power spectrum of the multi-doped ENZ tag is simulated and shown in Fig. 6e, where four reflection peaks indicate that four different dopants are all contained in the tag.

To map reflection spectra into the messages conveyed by the tag, we label the “presence” and the “absence” of a reflection peak as symbol “1” and “0”, respectively. For example, if all four dopants are used, the tag contains the information sequence of “1111”. Furthermore, the reflection spectrum of the doped ENZ tag allows to be digitally reconfigured. By removing different dopants, we use the tag to represent the information sequences of “0111”, “1011”, “1101”, and “1110”, which are illustrated in Fig. 6f–i, respectively. Generally, given a doped ENZ tag comprising *q* different dopants, we are able to sculpt 2^*q*^ kinds of possible dispersion curves, i.e., to generate 2^*q*^ types of messages, via choosing each dopant being retained or not.

## Discussion

In addition to the dynamic filtering and radio-frequency tagging, the method of dispersion coding of ENZ media may find applications in controlling optical nonlinear effect. Large optical nonlinearity enhancement was explored in ENZ media due to the significant enhancement and concentration of electromagnetic fields^14^. In this regard, the reconfigurable resonances due to photonic doping may also benefit the multiple wave mixing in ENZ media. The multi-band antenna based on ENZ meta-structures was investigated^47^, which can be tuned and switched based on the proposed scheme of dispersion coding. Importantly, assisted by suitable plasmonic materials for waveguide walls and by naturally occurring plasmonic materials, the concept of dispersion coding can be extended to higher frequency regions. The numerical results for such proposed implementation of dispersion coding in the terahertz and near-infrared regions are shown in Figs. S11, S12, respectively. Recently, the metasurfaces composed of periodically arranged elements were introduced to tailor the dispersion of the waveguide-emulated ENZ medium^48^. There are essential differences between the dispersion engineering approaches via metasurfaces and multiple photonic dopants. First, the dispersion of multi-doped ENZ medium is independent of the spatial arrangement of photonic dopants; in contrast, the spacing of constituent elements of metasurfaces has to be judiciously chosen. Second, the multiple resonant modes of a multi-band metasurface can be coupled; however, the magnetic resonances of multiple photonic dopants in an ENZ host are highly uncoupled. It is noteworthy that, besides realizing the frequency coding, it would be a promising direction to introduce the temporal coding^49^ or polarization coding^50^ into the multi-doped ENZ medium, owing to the strong interaction between the resonant photonic dopants and the incident waves.

In conclusion, we have demonstrated and validated the concept dispersion coding of ENZ media via multiple photonic dopants. The multiple dopants proved to serve as densely packed yet independent metamaterial bits on their magnetic resonances, which has rarely been predicted in conventional multi-resonance composites or cavities. Our analytical theory shows that the multi-doped ENZ medium can yield an effective permeability with a comb-like dispersion, and each resonance signature is dictated by one corresponding photonic dopant. Subsequently, we experimentally evidence the three-bit discrete control of the transmission spectrum of a waveguide-emulated ENZ medium by choosing specific dopant being retained or removed, and the measured result is in accordance with our theory. The proposed concept of dispersion coding could lead to exciting applications in a wide range of fields. As concrete examples, a discretely reconfigurable frequency-comb filter and a radio-frequency tag are devised, which are potentially applied for the signal processing and high-speed data storage and acquisition.

## Materials and methods

### Full-wave simulation

Simulations for two-dimensional structures (Figs. 2, 3, and 6b–i) were performed by using the RF module of the finite-element-method (FEM) commercial software COMSOL Multiphysics^®^ V5.0 (available at www.comsol.com). The 2D domain is discretized into triangular elements, where the maximum and minimum edge lengths of the elements are respectively 0.035*λ*_h_ (*λ*_h_ is the free-space wavelength at the highest operating frequency) and 0.011*λ*_h_. The stopping criterion is that the L2 norm of the relative residual of the matrix equation generated by FEM should be smaller than 1 × 10^−6^. Simulations for the three-dimensional structure (Fig. 5) were performed using the frequency domain solver of the commercial software CST STUDIO SUITE^®^ 2016 (available at www.3ds.com). The 3D domain is discretized into tetrahedral elements, where the maximum and minimum edge lengths of the elements are, respectively, 0.052*λ*_h_ and 0.016*λ*_h_, and the stopping criterion is met when the variation of the *S*-parameters is smaller than 0.01.

### Experiment setup and measurement

The metallic cavity to emulate the ENZ host in Fig. 3a was made of solid aluminum, processed by computer-numerical-controlled (CNC) metal machining technique. The feeding waveguides were constructed from Teflon (*ε*_r_ = 2.1 and dielectric loss tangent = 1 × 10^−3^) blocks tightly wrapped with copper tape. The ceramic blocks serving as dopants are processed via the line cutting technique into the predesigned sizes, while the copper strips fencing the dopants are obtained via the laser beam cutting technique. For the excitation and the reception of signal, two 50-Ω SMA connecters with 9-mm-length inner probes were armed at a distance of *λ*_*g*_/4 (*λ*_*g*_ is the guided wavelength in the substrate at the operating frequency 3 GHz) away from the end waveguide. The transmission coefficients were measured with a vector network analyzer Agilent N9917A.

## Supplementary information

Revised Supplementary Materials

## Data Availability

The simulation and experiment data that support the findings of this study are available from the corresponding author upon reasonable request.
