# Supplementary material for: Dispersion coding of ENZ media via multiple photonic dopants
Source: Light Sci Appl. 2022 Jul 6;11:207. doi: 10.1038/s41377-022-00892-8 (PMC9259670; doi:10.1038/s41377-022-00892-8)
Supplement: Supplementary file 1 — Revised Supplementary Materials [file 41377_2022_892_MOESM1_ESM.docx]

**Supplementary Information for**

**Dispersion coding of ENZ media via multiple photonic dopants**

**Ziheng Zhou^1^**^†^**,** **Hao Li****^1^**^†^**, Wangyu Sun^1^, Yijing He^1^, Iñigo Liberal^2^,**

**Nader Engheta^3^, Zhenghe Feng^1^, Yue Li^1,^***

^1^Department of Electronic Engineering, Tsinghua University, Beijing 100084, China

^2^Department of Electrical and Electronic Engineering, Public University of Navarre, Pamplona 31006, Spain

^3^Department of Electrical and Systems Engineering, University of Pennsylvania, Philadelphia, PA 19104, USA

^†^These authors contributed equally to this work

*Corresponding author

**Official email addresses of all authors:**

Ziheng Zhou, e-mail: zhouzh17@mails.tsinghua.edu.cn

Hao Li, e-mail: h-li18@mails.tsinghua.edu.cn

Wangyu Sun, e-mail: swy19@mails.tsinghua.edu.cn

Yijing He, e-mail : heyj16@mails.tsinghua.edu.cn

Iñigo Liberal, e-mail: inigo.liberal@unavarra.es

Nader Engheta, e-mail: engheta@ee.upenn.edu

Zhenghe Feng, e-mail: fzh-dee@tsinghua.edu.cn

Yue Li (corresponding author), e-mail: lyee@tsinghua.edu.cn

**This file includes:**

Supplementary Notes 1, 2

Supplementary Figures 1-12

References

**Supplementary Note 1: Derivation of Eq. (2) in the main text.**

The effective permeability *μ*_eff_ of the doped ENZ medium can be derived via averaging the magnetic flux over regions of the dopants and the ENZ host, which is generally formulated as^3^:

 (S1)

, where *A* is the cross-sectional area of the whole doped medium, *A_d_* (*d* = 1, 2, 3…) are the cross-sectional areas of the dopants included, while *ψ^d^* (*d* = 1, 2, 3…) denote the magnetic field in the dopants normalized to unity on their boundaries. Specifically, for the dopant with a rectangular cross-sectional area of *l_d_* _­_×*w_d_* (refer to Figure S1), the magnetic field distribution *ψ^d^* can be accurately solved via Green’s function technique, and the result is presented in our previous work^4^:

$\psi^{d}(x,y)=1+\sum_{m=1,n=1}^{+\infty} \varepsilon_{d}\frac{\omega^{2}}{c^{2}}\frac{4((-1)^{m}-1)((-1)^{n}-1)}{\pi^{2}mn}\frac{\cos(m\pi x/l_{d})\cos(n\pi y/w_{d})}{\left(m\pi/l_{d}\right)^{2}+\left(n\pi/w_{d}\right)^{2}-\varepsilon_{d}(\omega/c)^{2}}$ (S2)

, where *ε_d_* is the relative permittivity of the dielectric dopant. Substituting the expression of *ψ^d^* into Eq. S1, we readily arrive at the effective permeability of the multi-doped ENZ medium:

$\mu_{\text{eff}}(\omega)=\text{1+}\frac{1}{l w}\sum_{d=1} \sum_{m=1,n=1}^{+\infty} \frac{4l_{d}w_{d}((-1)^{m}-1)^{2}((-1)^{n}-1)^{2}}{\pi^{4}m^{2}n^{2}}\frac{\varepsilon_{d}\left(\omega/c\right)^{2}}{\left(m\pi/l_{d}\right)^{2}+\left(n\pi/w_{d}\right)^{2}-\varepsilon_{d}\left(\omega/c\right)^{2}}$ (S3)

, where *l*×*w* is the area of the ENZ medium. As is expressed by Eq. S3, each dopant impacts the permeability dispersion of the whole ENZ region by inserting the poles pinpointed at:

$\omega_{d,m,n}=\frac{c}{\sqrt{\varepsilon_{d}}}\sqrt{\left(\pi m\text{/}l_{d}\right)^{2}+\left(\pi n\text{/}w_{d}\right)^{2}}$ (S4)

, which actually are the eigenfrequencies the transverse-magnetic TM*_m_*_,_*_n_* (with respective to the out-of-plane axis) mode of the dopants. Here, the plasmonic frequency *ω*_p_, is designed near the fundamental resonance modes (*m* = *n* =1) of the dopants and far below the frequencies of higher-order modes. Hence, we truncate the infinite series in Eq. S2 by the dominant term with indexes *m*, *n* = 1, and therefore the effective permeability (Eq. S3) near *ω*_p_ reduces to a much explicit form:

$\mu_{\text{eff}}(\omega)\approx\text{1+}\sum_{d=1} \frac{64l_{d}w_{d}}{\pi^{4}l w}\frac{\omega^{2}}{\omega_{d,1,1}^{2}-\omega^{2}}$ (S5)

, where the expression for frequencies *ω_d_*_,1,1_ has been applied using Eq. S4 with *m* = *n* = 1. Finally, by using the notation:

**$\omega_{d}\equiv\omega_{d,1,1}$** (S6)

we arrive at the Eq. (2) in the main text.

**Supplementary Note 2: Transfer matrix method to calculate the transmission coefficient of the multi-doped ENZ medium.**

To quantitatively analyze how multi-doped ENZ medium modulates the wave propagation, we model the doped ENZ slab in Fig. 1b of main text by the transmission matrix method^5^:

$T=\left[\begin{matrix} \cos(\sqrt{\varepsilon_{\text{h}}\mu_{\text{eff}}}\omega l/c) & -i\sqrt{\mu_{\text{eff}}/\varepsilon_{\text{h}}}\sin(\sqrt{\varepsilon_{\text{h}}\mu_{\text{eff}}}\omega l/c) \\ -i\sqrt{\varepsilon_{\text{h}}/\mu_{\text{eff}}}\sin(\sqrt{\varepsilon_{\text{h}}\mu_{\text{eff}}}\omega l/c) & \cos(\sqrt{\varepsilon_{\text{h}}\mu_{\text{eff}}}\omega l/c) \end{matrix}\right]$ (S7)

, where *μ*_eff_ is the effective relative permeability of the doped ENZ slab given by Eq. S3. A *e*^-^*^i^*^ωt^ time convention is assumed and omitted hereafter. Then, the transmission coefficient S_21_ of the doped ENZ slab connected to the outside air-filled plate waveguides can be formally derived as:

$\begin{matrix} \text{S}_{21}=\frac{2}{\sum_{m,n} T_{m,n}} =\frac{2}{2\cos(\sqrt{\varepsilon_{\text{h}}\mu_{\text{eff}}}\frac{\omega l}{c})-i(\sqrt{\frac{\varepsilon_{\text{h}}}{\mu_{\text{eff}}}}+\sqrt{\frac{\mu_{\text{eff}}}{\varepsilon_{\text{h}}}})\sin(\sqrt{\varepsilon_{\text{h}}\mu_{\text{eff}}}\frac{\omega l}{c})} \\ \approx\frac{2}{2-\varepsilon_{\text{h}}\mu_{\text{eff}}(\omega l/c)^{2}-i(\varepsilon_{\text{h}}+\mu_{\text{eff}})\omega l/c} \end{matrix}$ (S8)

Considering the ENZ condition *ε*_h_ ≈ 0, Taylor expansions of sine and cosine functions (sin(*x*) ≈ *x*, cos(*x*) ≈ 1− *x*^2^/2) for the small argument can be used to simply the expression.

**Supplementary Figures**

**
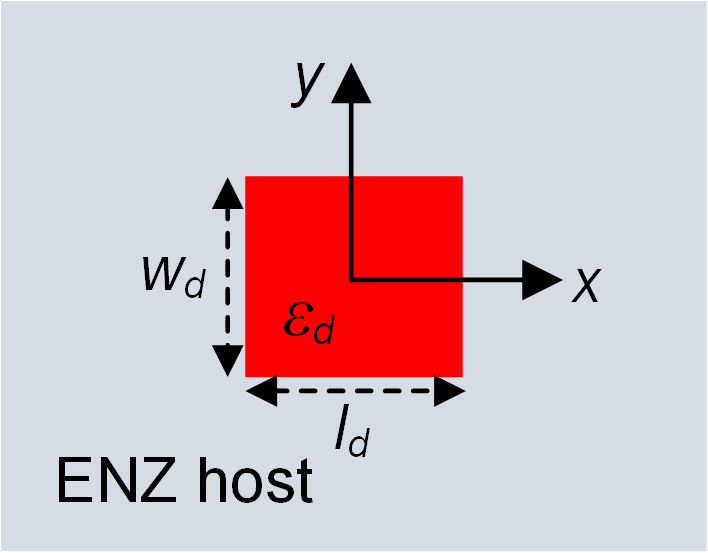
**

**Figure S1|** **2D** **configuration of doped ENZ media.** Two-dimensional schematic of a rectangular dopant placed in a 2D ENZ host.





**Figure S2| The dB values of the transmission amplitudes shown in the Fig. 1d of the main text.**


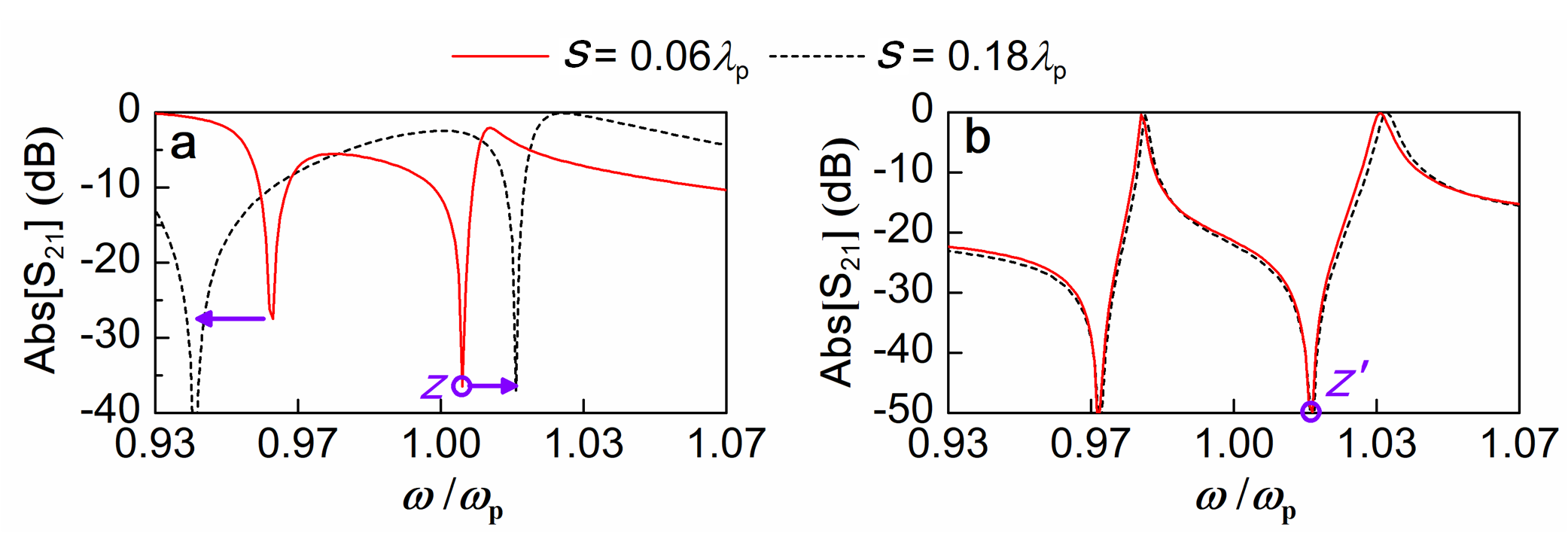


**Figure S3|** **The** **dB values of the results in Figs. 2c and 2d.** (a) The dB values of the transmission amplitudes shown in the Fig. 2c of the main text. (b) The dB values of the transmission amplitudes shown in the Fig. 2d of the main text.





**Figure S4|** **The** **dB values of the results in Figs. 3b and 3c.** (a) The dB values of the transmission amplitudes shown in the Fig. 3b of the main text. (b) The dB values of the transmission amplitudes shown in the Fig. 3c of the main text.


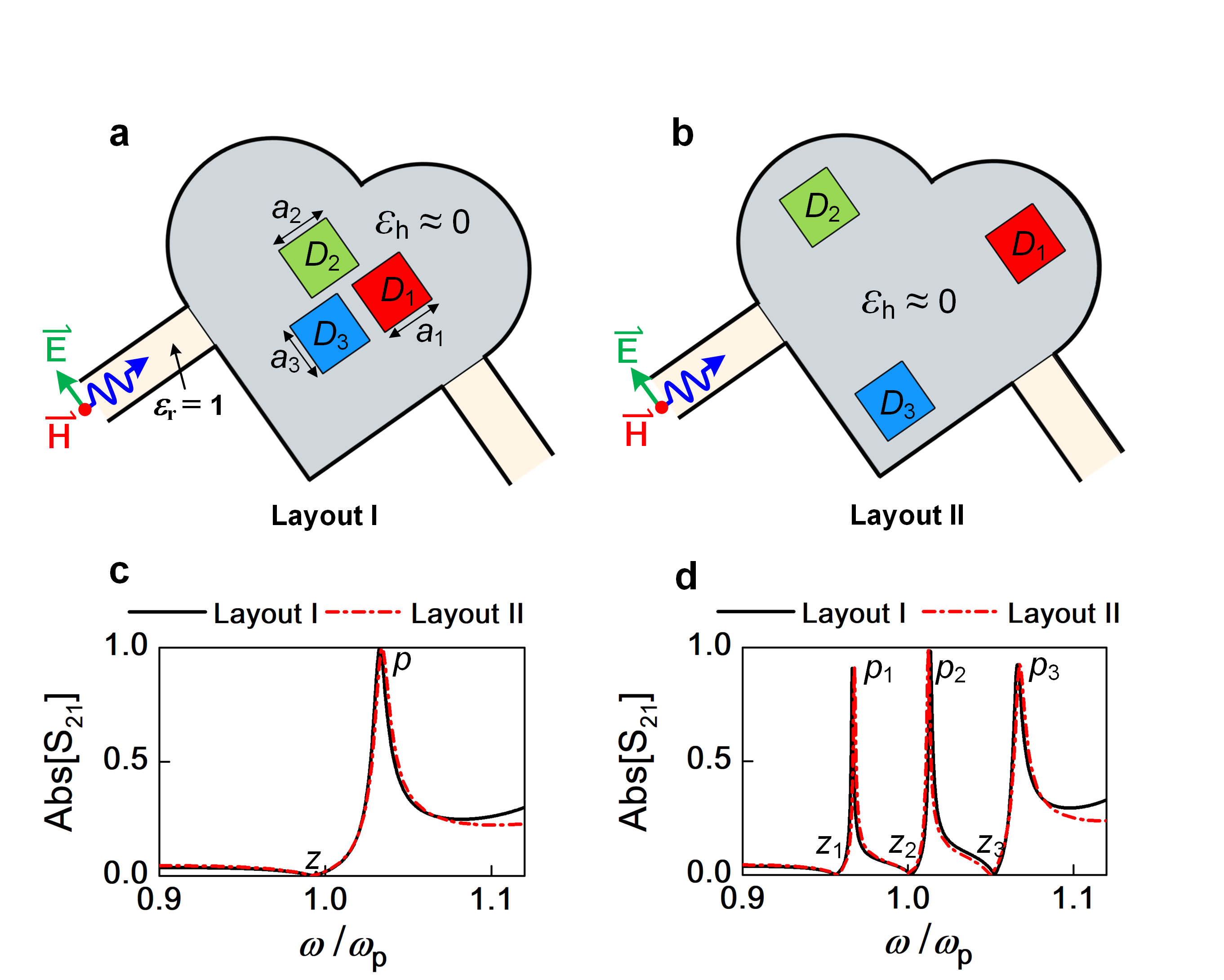


**Figure S5|** **ENZ medium comprising photonic dopants under different arrangements.** Three square dielectric resonators with a relative permittivity of 37 and side lengths *a*_1_, *a*_2_, *a*_3_ are embedded in an ENZ medium, and they are arranged by different layouts (**a**) and (**b**). Two waveguides filled with air are connected with the ENZ cavities for the transmission response testing, under the incident wave polarized with the magnetic field along the out-of-plane axis. (**c**) Simulated transmission spectrum, for the resonators being identically sized with *a*_1_ = *a*_2_ = *a*_3_ = 0.117*λ*_p_ (*λ*_p_ is the free-space wavelength at the plasma frequency *ω*_p_ of the ENZ medium). (**d**) Simulated transmission spectrum for the resonators being differently sized with *a*_1_ = 0.122*λ*_p_, *a*_2_ = 0.116*λ*_p_, and *a*_3_ = 0.110*λ*_p_.


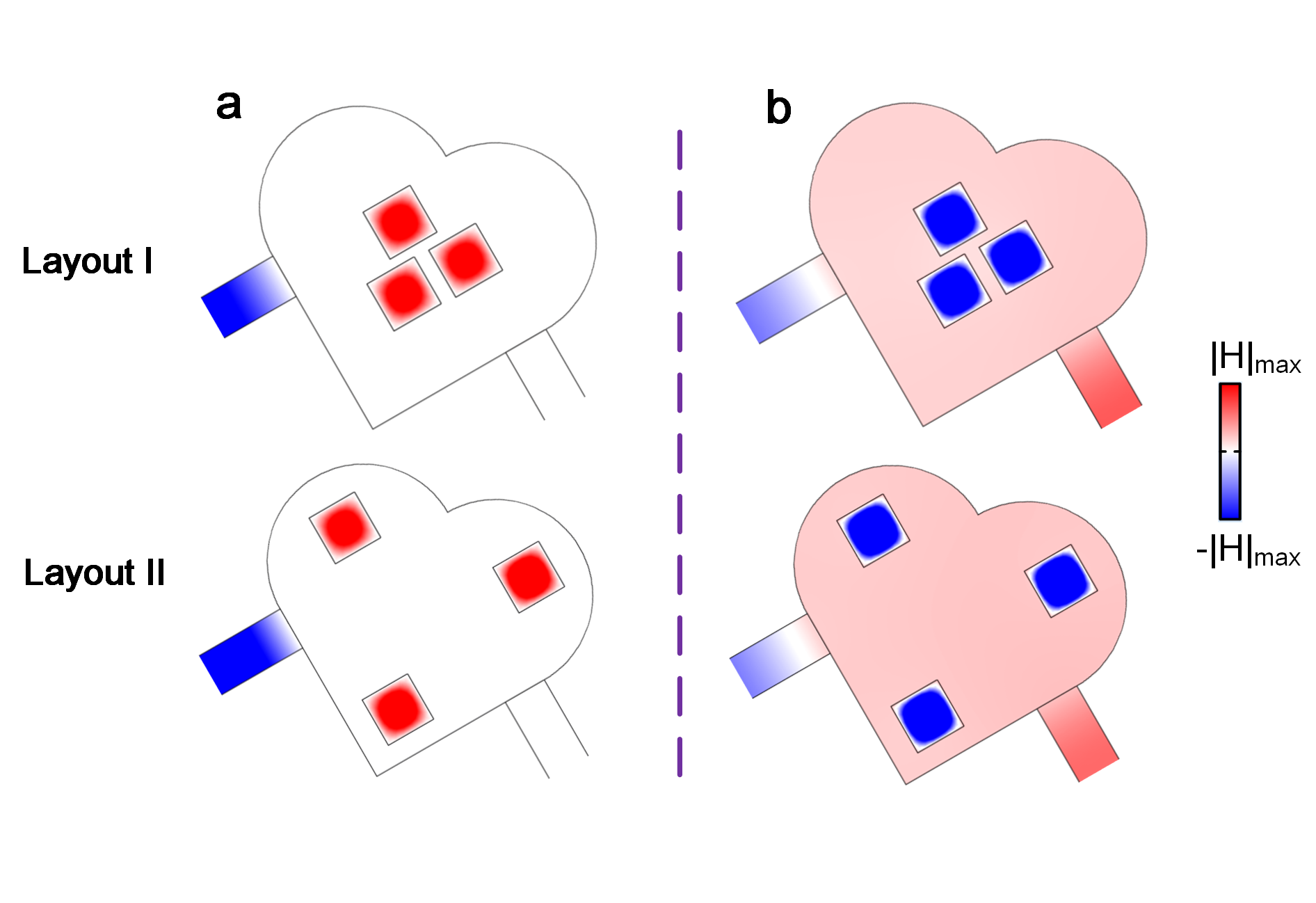


**Figure S6|** **Simulated magnetic field distribution for the case of triple identical dopants.** (**a**) Snapshots of the magnetic field at the transmission zeros *z* (marked in Figure S5c) for layout I (Figure S5a) and layout II (Figure S5b). (**b**) Snapshots of the magnetic field at the transmission peaks *p* (marked in Figure S5c) for layout I (Figure S5a) and layout II (Figure S5b).


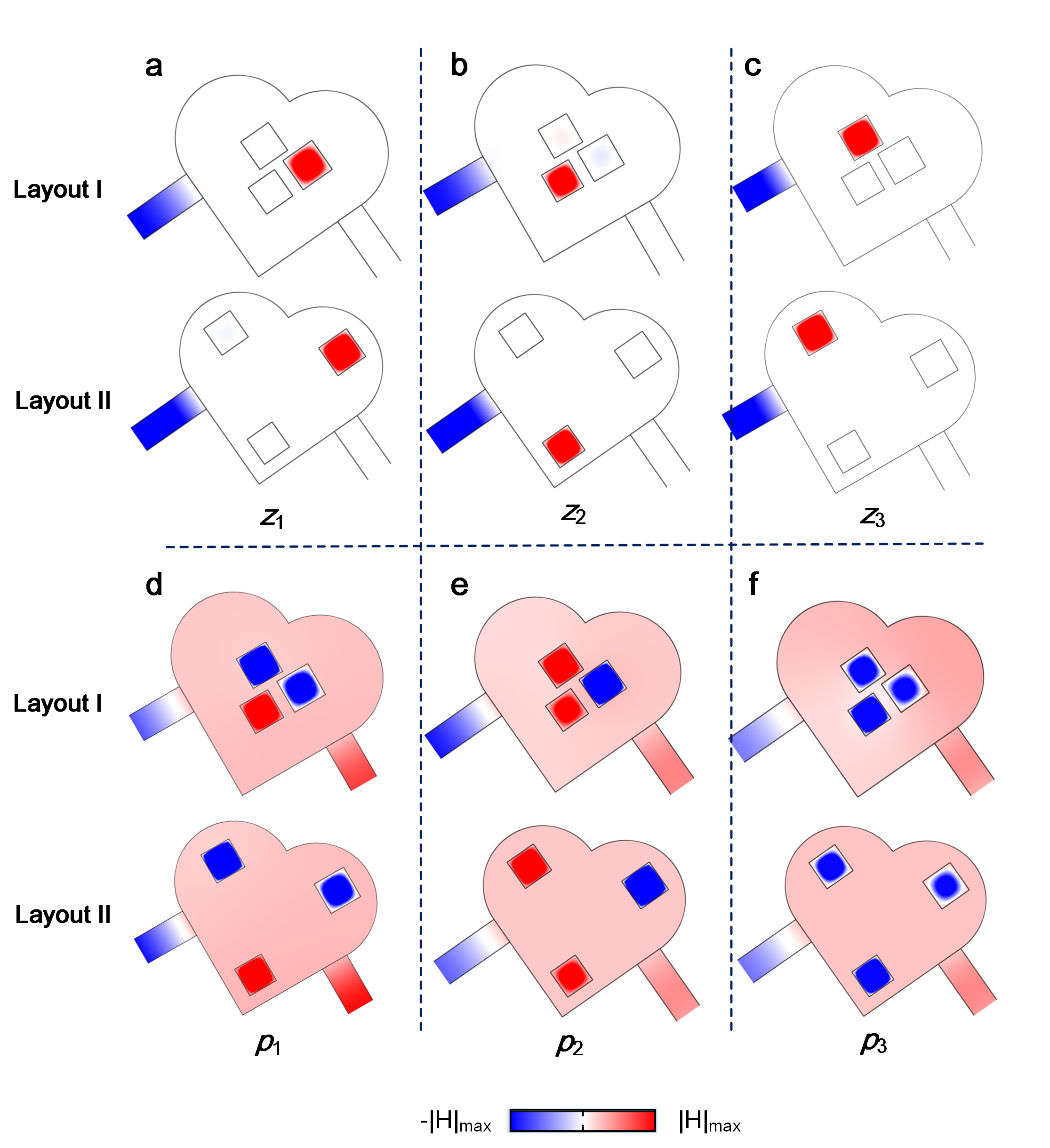


**Figure S7|** **Simulated magnetic field distribution for the case of triple differently-sized dopants.** (**a**) - (**c**) are, respectively, snapshots of the magnetic field at the transmission zeros *z*_1_, z_2_, and z_3_ (marked in Figure S5d) for layout I (Figure S5a) and layout II (Figure S5b). (**d**) - (**f**) are, respectively, snapshots of the magnetic field at the transmission peaks *p*_1_, *p*_2_, and *p*_3_ (marked in Figure S5d) for layout I (Figure S5a) and layout II (Figure S5b).


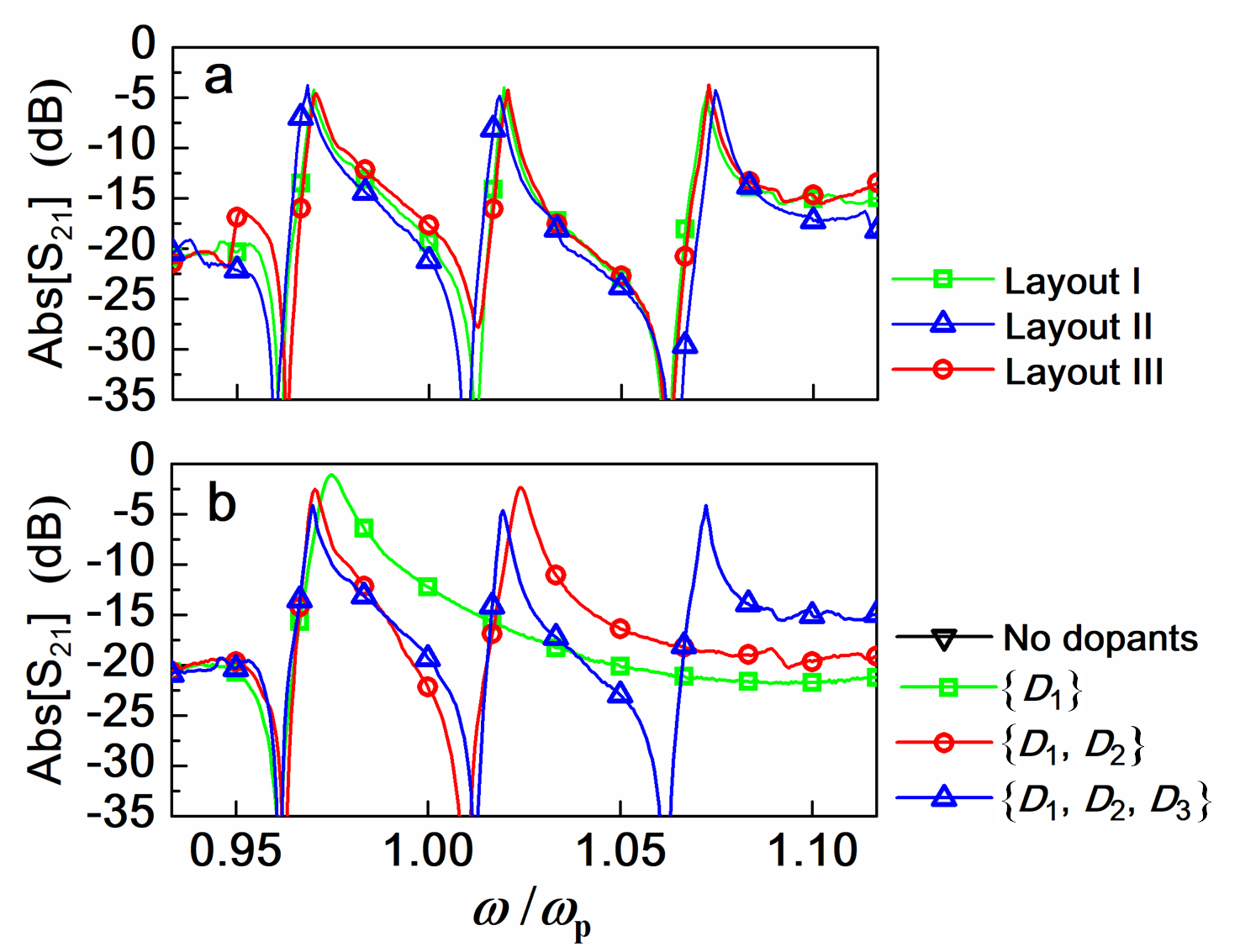


**Figure S8 | The** **dB values of the results in Figs. 4b and 4d.** (a) The dB values of the transmission amplitudes shown in the Fig. 4b of the main text. (b) The dB values of the transmission amplitudes shown in the Fig. 4d of the main text.


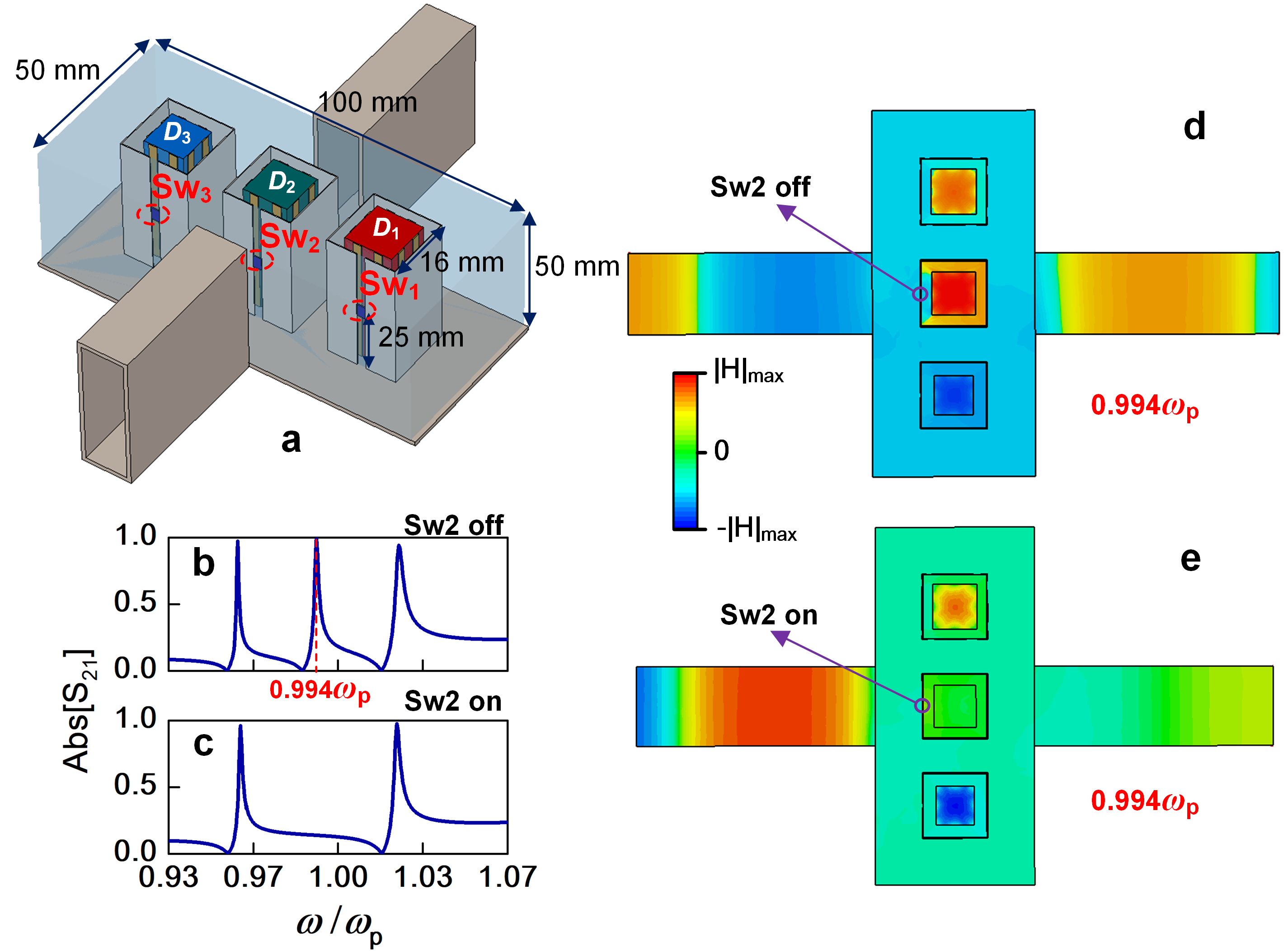


**Figure S9|** **Dynamical control of photonic doping**. (**a**) Proposed 3D structure of the switchable doping system. The upper and side walls of the doped metallic cavity are not shown here. (**b**) and (**c**) are, respectively, the simulated transmission spectrums for the switch Sw_2_ being turned off and on. (**d**) and (**e**) are the simulated snapshots of magnetic field distributions at 0.994 *ω*_p_ for the switch Sw_2_ being turned off and on. If the switch is closed on, the magnetic field within the dopant diminishes to zero.





**Figure S10| The dB values of the results in Figs. 5c and 5d.** (a) The dB values of the transmission amplitudes shown in the Fig. 5c of the main text. (b) The dB values of the transmission amplitudes shown in the Fig. 5d of the main text.


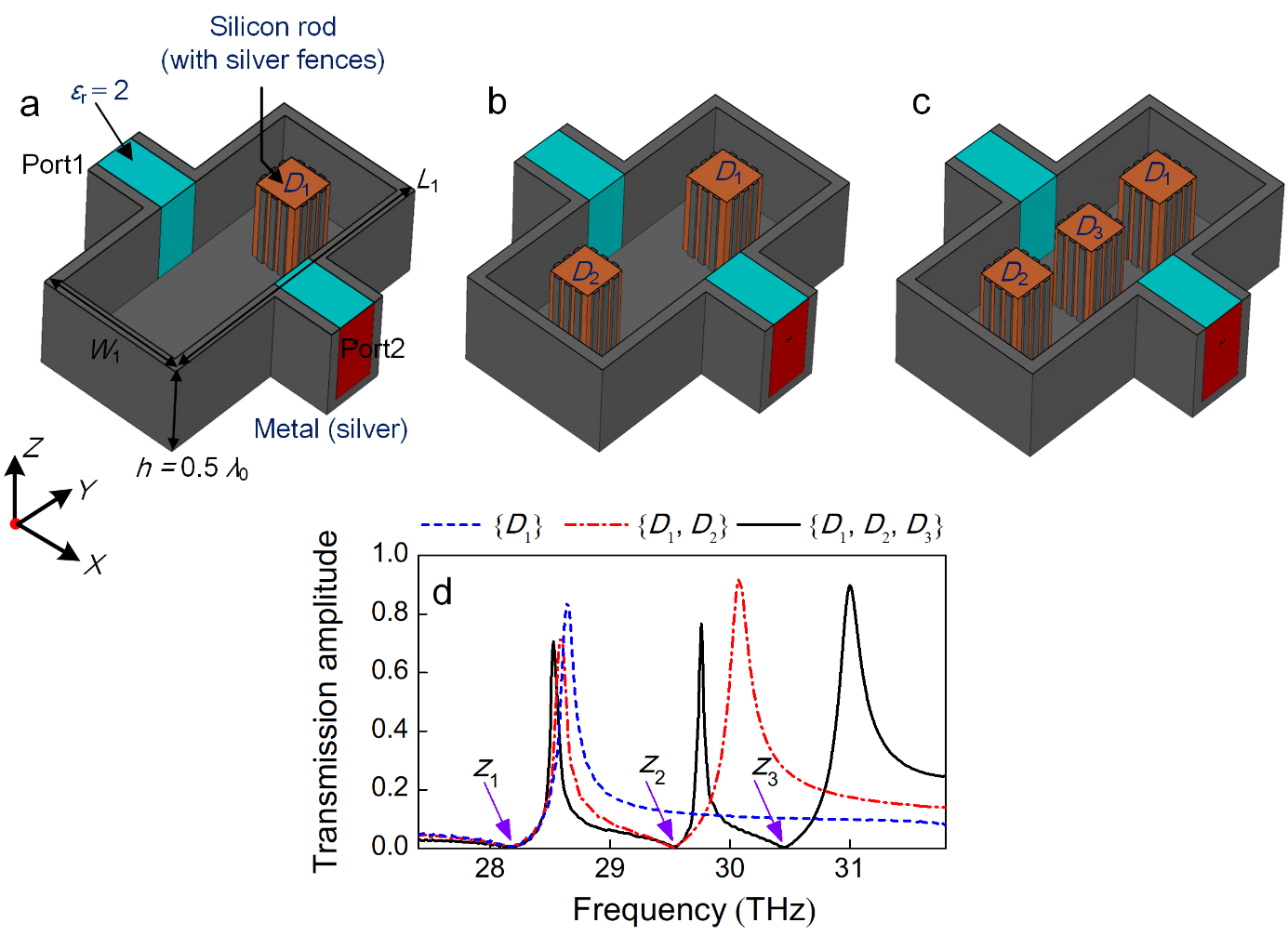


**Figure S11| Implementation of dispersion coding in the terahertz region.** (a), (b), (c) are the configurations waveguide-emulated ENZ media containing one, two, and three silicon photonic dopants (*ε*_r_ = 11.9 and dielectric loss tangent of 2e-4). The height of the waveguide *h* is chosen to be the half wavelength at the effective plasmonic frequency *f*_0_ = 29.5 THz. To show the inner details, the upper claddings of waveguides are not shown in the figures. The cross-sectional area of the waveguide cavity on *xy* plane is *L*_1_ × *W*_1_ =13 μm × 6 μm, and photonic dopants *D*_1_, *D*_2_, and *D*_3_ are square rods with side lengths of cross-sectional shapes being 2.08 μm, 1.92 μm, and 1.86 μm, respectively. The waveguides are wrapped by silver, whose permittivity is described Drude model with the plasma frequency of 2.18 PHz and the damping frequency of 4.35 THz^1^. The fences around photonic dopants are also made of silver. (d) Simulated transmission spectra for cases (a), (b), and (c).


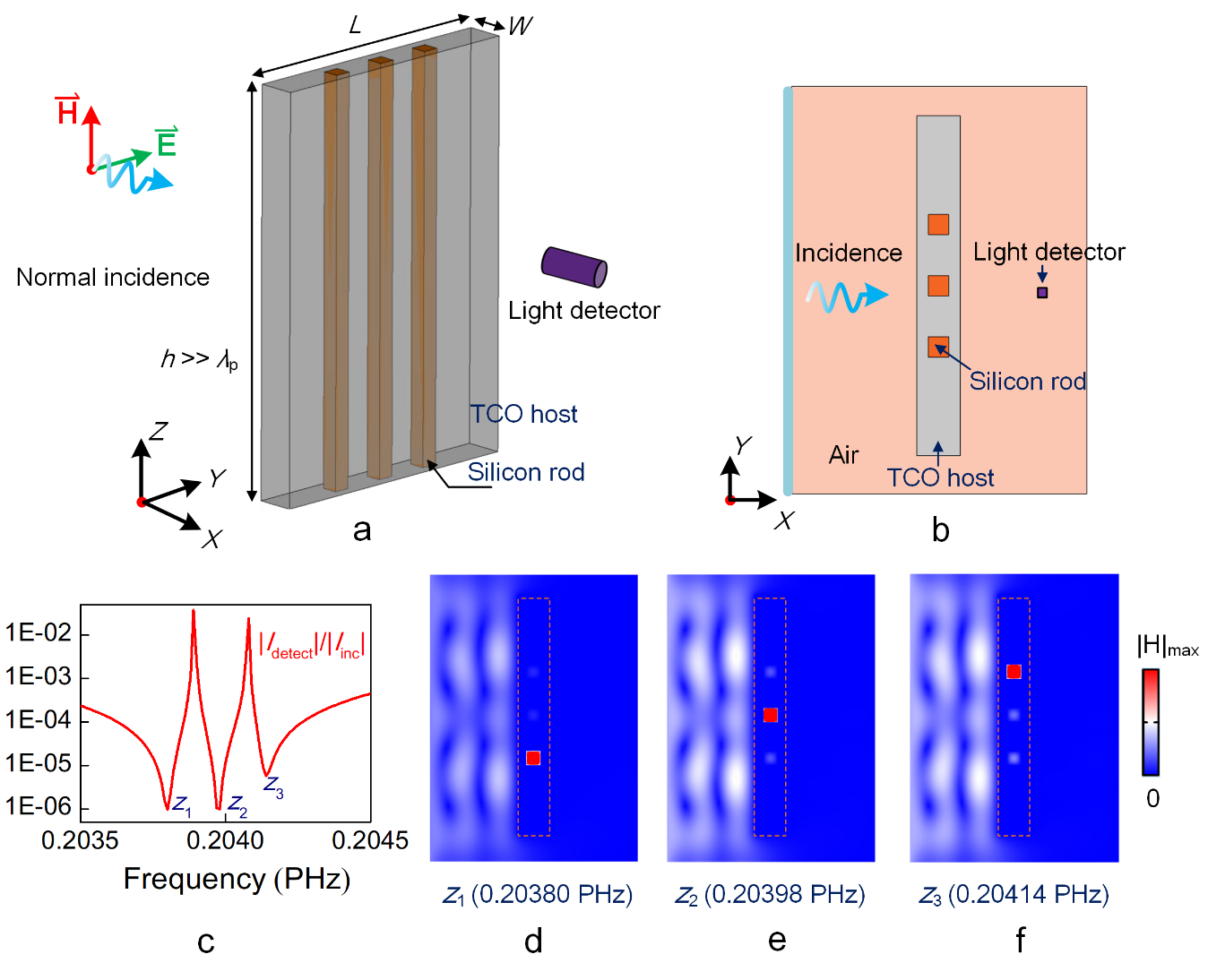


**Figure S12|** **Implementation of dispersion coding in the infrared region.** (a) Configuration of a transparent conductive oxide (TCO)^2^ material slab (with the plasma frequency *f*_p_ set as 0.204 PHz and the damping frequency set as 0.38 THz) containing three silicon photonic dopants under transmittance test and (b) its two-dimensional representation. The cross-sectional area of TCO slab in the *xy* plane is *L* × *W* = 5 μm × 0.64 μm (3.40*λ*_p_ × 0.435*λ*_p_, *λ*_p_ is the free-space wavelength at *f*_p_ = 0.204 PHz), and the cross-sectional sizes of three dopants are 0.2040*λ*_p_ ×0.2033*λ*_p_, 0.2040*λ*_p_ ×0.2040*λ*_p_, and 0.2040*λ*_p_ ×0.2047*λ*_p_, respectively. (c) Numerical simulation result of detected light intensity over the incident light intensity. (d), (e), (f) are magnetic field magnitude distributions at three transmission zeros *z*_1_, *z*_2_, and *z*_3_, respectively.

**References**

1. Ordal, M. A. *et al*. Optical properties of fourteen metals in the infrared and far infrared: Al, Co, Cu, Au, Fe, Pb, Mo, Ni, Pd, Pt, Ag, Ti, V, and W. *Applied Optics* **24**, 4493-4499 (1985)
2. Naik, G. V., Kim, J., & Boltasseva, A. Oxides and nitrides as alternative plasmonic materials in the optical range. *Optical Materials Express* **1**, 1090-1099 (2011).
3. Liberal, I. *et al*. Photonic doping of epsilon-near-zero media. *Science* **355**, 1058-1062 (2017).
4. Zhou, Z. H. *et al*. Substrate-integrated photonic doping for near-zero-index devices. *Nature Communications* **10**, 4132 (2019).
5. Pozar, D. M. *Microwave Engineering*, 4th Ed*.* (John Wiley & Sons, Inc., New York, 2012).
